# *Equisetum arvense* Inhibits Alveolar Bone Destruction in a Rat Model with Lipopolysaccharide (LPS)-Induced Periodontitis

**DOI:** 10.1155/2022/7398924

**Published:** 2022-12-30

**Authors:** Fumie Shiba, Hisako Furusho, Takashi Takata, Rika Shimizu, Mutsumi Miyauchi

**Affiliations:** ^1^Research and Development Headquarters, Earth Corporation Ltd, Hyogo 678-0192, Japan; ^2^Department of Oral and Maxillofacial Pathobiology, Graduate School of Biomedical and Health Science, Hiroshima University, Hiroshima 734-8553, Japan; ^3^Shunan University, Yamaguchi 745-8566, Japan

## Abstract

**Background and Aims:**

*Equisetum arvense* extract (EA) exerts various biological effects, including anti-inflammatory activity. The effect of EA on alveolar bone destruction has not been reported; therefore, we aimed to determine whether EA could inhibit alveolar bone destruction associated with periodontitis in a rat model in which periodontitis was induced using lipopolysaccharide from *Escherichia coli* (*E*. *coli*-LPS).

**Methods:**

Physiological saline or *E*. *coli*-LPS or *E*. *coli*-LPS/EA mixture was topically administered into the gingival sulcus of the upper molar region of the rats. After 3 days, periodontal tissues of the molar region were collected. Immunohistochemistry was performed for cathepsin K, receptor activator of NF-*κ*B ligand (RANKL), and osteoprotegerin (OPG). The cathepsin K-positive osteoclasts along the alveolar bone margin were counted. EA effects on the expression of the factors regulating osteoclastogenesis in osteoblasts with *E*. *coli*-LPS-stimulation were also examined *in vitro*.

**Results:**

Treatment with EA significantly reduced the number of osteoclasts by decreasing the RANKL-expression and increasing OPG-expression in the periodontal ligament in the treatment group compared to the *E*. *coli*-LPS group. The *in vitro* study showed that the upregulation of p-I*κ*B kinase *α* and *β* (p-IKK*α*/*β*), p-NF-*κ*B p65, TNF-*α*, interleukin-6, and RANKL and downregulation of semaphorin 3A (Sema3A), *β*-catenin, and OPG in the osteoblasts with *E*. *coli*-LPS-stimulation improved with EA-treatment.

**Conclusion:**

These findings demonstrated that topical EA suppressed alveolar bone resorption in the rat model with *E*. *coli*-LPS-induced periodontitis by maintaining a balance in RANKL/OPG ratio via the pathways of NF-*κ*B, Wnt/*β*-catenin, and Sema3A/Neuropilin-1. Therefore, EA possesses the potential to prevent bone destruction through inhibiting osteoclastogenesis attributed to cytokine burst under plaque accumulation.

## 1. Introduction

Periodontitis is an inflammatory/infectious disease of the periodontal tissues caused by dental plaque at the interface between the teeth and the gingiva. Dental plaque contains bacterial components, such as lipopolysaccharide (LPS), which promotes osteoclast differentiation through the production of proinflammatory factors, such as tumor necrosis factor-*α* (TNF-*α*), interleukin-1 (IL-1), and prostaglandin from the osteoblasts [1]. LPS promotes osteoclastogenesis not only indirectly through inflammatory cytokine production from the osteoblasts but also directly through the production of the receptor activator of nuclear factor-*κ*B ligand (RANKL), an osteoclastogenic factor. Moreover, LPS also suppresses the expression of osteoprotegerin (OPG), a decoy receptor for RANKL, thereby increasing the RANKL/OPG ratio and finally causing osteoclast formation and maturation, which consequently leads to bone resorption [2–5].

Hence, elimination of plaque accumulation, cytokine burst, overproduction of RANKL, and decreased production of OPG attributed to LPS are essential for preventing alveolar bone destruction associated with periodontitis.

Currently, plaque removal and the creation of an oral environment resistant to plaque adhesion form the basis of prevention and treatment of periodontitis [6]. Regular brushing is the most effective means of preventing periodontitis by maintaining good oral hygiene [7]. However, individuals who are disabled or bedridden tend to have some difficulty in brushing satisfactorily and are prone to severe periodontitis. Therefore, the development of a supplementary oral care method to prevent the destruction of alveolar bone associated with periodontitis that can be easily applied by anyone daily in addition to brushing is desired.

*Equisetum arvense* (*E. arvense*) is a species of fern that is widespread in the northern hemisphere, especially in Europe and North and Central America [8, 9]. Its medicinal properties have been explored since its use by the ancient Greeks and Romans for treating wounds [10, 11]. *E. arvense* extract (EA) has various potential pharmacological properties, including antiseptic, antiseborrheic, diuretic, anti-inflammatory, antioxidant, hepatoprotective, vasoprotective, vasorelaxant, and antinociceptive [8–14]. Moreover, *E. arvense* contains secondary metabolites, such as quercetin, kaempferol, luteolin, apigenin, oleanolic acid, betulinic acid, and ursolic acid [13, 15, 16], which positively affect bone formation by osteoblasts [17–26]. Moreover, among known plants, *E. arvense* contains the highest concentration of silica [27, 28]. However, the mechanism of EA's effect on bone is poorly understood. Moreover, the effects of EA in alveolar bone destruction attributed to periodontitis are unclear.

Previously, by using a rat model of LPS-induced periodontitis, we confirmed that administration of EA into the gingival sulcus markedly reduced immunoexpression of TNF-*α* at the junctional epithelium [29]. TNF-*α* promotes the increase of osteoclast progenitor cells *in vivo* [30–33], and inhibition of human osteoclastogenesis *in vitro* by EA has also been reported [10, 34]; therefore, we suggested that EA could inhibit alveolar bone destruction associated with periodontitis.

In this study, we aimed to clarify whether EA could inhibit alveolar bone destruction associated with periodontitis using a rat model in which periodontitis was induced using LPS from *Escherichia coli* (*E*. *coli*-LPS). Furthermore, we investigated whether EA is useful in the prevention and treatment of periodontal and other bone destructive diseases and also determined the mechanism of inhibitory effects of EA on bone destruction.

## 2. Materials and Methods

### 2.1. Reagents

EA (extracted from whole *E. arvense* with 50% 1,3-butylene glycol solution) was purchased from Maruzen Pharmaceuticals Co., Ltd. (Hiroshima, Japan). LPS from *Escherichia coli* (*E*. *coli*-LPS: B6) was purchased from Sigma-Aldrich (Saint Louis, MO, USA).

### 2.2. Cell Culture

ST2 cells, cloned stromal-cell lines from mouse bone marrow, were cultured in the alpha minimum essential medium (*α*-MEM) (Invitrogen, MA, USA), containing 10% fetal bovine serum (Thermo Fisher Scientific, MA, USA) and supplemented with 100 U/mL of penicillin and 100 *μ*g/mL of streptomycin. Cells were maintained in a humidified incubator at 37°C with 5% CO_2_.

### 2.3. Quantitative Real-Time Polymerase Chain Reaction (PCR)

ST2 cells (8 × 10^5^ cells/3 mL/plate) were seeded into a Φ 6 cm plate for 1 day. After incubation in the fresh medium for 1 day, the cells were further incubated with *E*. *coli*-LPS (1.0 *μ*g/mL) and EA (3 *μ*g/mL) for 2, 12, and 24 hours to analyze the mRNA involved in osteoclastogenesis, including interleukin-6 (IL-6), TNF-*α*, interleukin-1*β* (IL-1*β*), RANKL, OPG, and semaphorin 3A (Sema3A). Total RNA was extracted from ST2 cells and purified by using the RNeasy Mini Kit (QIAGEN, Hilden, Germany). After cDNA synthesis using ReverTra Ace® (Toyobo Co., Ltd., Osaka, Japan) according to the manufacturer's protocol, the Applied Biosystems® StepOne™ Real-time PCR System (Thermo Fisher Scientific, MA, USA) was used with the FastStart Essential DNA Green Master (Roche Diagnostics GmbH, Mannheim, Germany) according to the manufacturer's protocol. Reactions were conducted for up to 45 cycles with denaturing at 95°C, annealing at 57°C, and extension at 72°C. The primers used for this system were as follows: mouse TNF-*α*, 5′-ATGAGCACAGAAAGCATGATC-3′ (sense) and 5′-TACAGGCTTGTCACTCGAATT-3′ (antisense); mouse IL-6, 5′-TTACACATGTTCTCTGGGAAATCGT-3′ (sense) and 5′-TGGTAGCATCCATCATTTCTTTGT-3′ (antisense); mouse IL-1*β*, 5′-AGAGAGCCTGTGTTTTCCTCCTTG-3′ (sense) and 5′-GCTTCAATGAAAGACCTCAGTGCAG-3′ (antisense); mouse RANKL, 5′-GCACACCTCACCATCAATGC-3′ (sense) and 5′-GTCTGTAGGTACGCTTCCCG-3′ (antisense); mouse OPG, 5′-CAGAGACTAATAGATCAAAGGCA-3′ (sense) and 5′-ATGAAGTCTCACCTGAGAAGAAC-3′ (antisense); mouse Sema3A, 5′-CAGTTGGGGAACTCTGGCTC-3′ (sense) and 5′-CAGGTTGCCCTCTGGGTTAG-3′ (antisense); glyceraldehyde 3-phosphate dehydrogenase (GAPDH) as an internal standard, 5′-TGAACGGGAAGCTCACTGG-3′ (sense) and 5′- TCCACCACCCTGTTGCTGTA-3′ (antisense).

### 2.4. ELISA System

ST2 cells (2.6 × 10^5^ cells/mL/well) were seeded into a 12 well plate for 1 day. After incubation in the fresh medium for 1 day, the cells were further incubated with *E*. *coli*-LPS (1.0 *μ*g/mL) and EA (3 *μ*g/mL) for 3 hours or 48 hours to analyze the production of RANKL or OPG. The cell supernatant was corrected and analyzed by the Quantikine® ELISA Mouse TRANCE/RANKL/TNFSF11 Immunoassay (#MTR00, R&D Systems, MN, USA) or the Quantikine® ELISA Mouse Osteoprotegerin/TNFRSF11B kit (#MOP00, R&D Systems, MN, USA) according to the manufacturer's protocol.

### 2.5. Western Blotting

ST2 cells (3 × 10^5^ cells/mL) were cultured in a Φ 6 cm plate and stimulated with *E*. *coli*-LPS (1 *μ*g/mL), EA (3 *μ*g/mL). Western blotting was performed as described previously by Kudo et al. [35]. In brief, cell pellets were resuspended in ice-cold lysis buffer. Proteins were separated by SDS-PAGE, electro-blotted onto nitrocellulose membrane, and were visualized by the ECL western blotting detection system (GE Healthcare, UK) (Amersham, Piscataway, NJ, USA). The following antibodies from Cell Signaling Technology were used as primary antibodies: p-NF-*κ*B p65 (#3033; 1 : 1,000), NF-*κ*B p65 (#8242; 1 : 1,000), p-p38mitogen-activated protein kinase (p-p38 MAPK) (#4511; 1 : 1,000), p38 MAPK (#8690; 1 : 1,000), p-SAPK/JNK (#4668; 1 : 1,000), t-SAPK/JNK (#9258; 1 : 1,000), p-IKK*α*/*β* (S176/180) (#2697S; 1 : 1,000), IKK*β* (#2678; 1 : 1,000), and *β*-actin (#A2228; 1 : 8,000; Sigma-Aldrich). The following antibody from GeneTex was used as the primary antibody: beta catenin antibody [*N*1*N*2-2], *N*-term (#GTX101435; 1 : 1,000).

### 2.6. Animal Experiments

The experimental protocol described as follows was approved by the Animal Care Committee of Hiroshima University (Permit Number: A20–32). A total of 11, 8-week-old male Wistar rats weighing 330 ± 11.9 g (range: 315–355 g) (Charles River Japan, Inc., Yokohama, Japan) were housed in a specific-pathogen-free facility in 12 hour light-dark cycles with access to water and food ad libitum and kept at a constant ambient temperature and humidity (22°C, 50 ± 5% relative humidity).

During the experiment, the rats were anesthetized using an intraperitoneal injection, comprising a mixture of three anesthetics (4.56 mL/kg/animal): medetomidine hydrochloride (0.03 mg/mL, Dorbene® vet, Kyoritsu Seiyaku Co., Tokyo, Japan), midazolam (0.4 mg/mL, Midazolam Injection 10 mg, Sandoz K. K., Tokyo, Japan), and butorphanol tartrate (0.5 mg/mL, Vetorphale®, Meiji Seika Pharma Co., Ltd., Tokyo, Japan) which were diluted with saline (OTSUKA NORMAL SALINE, Otsuka Pharmaceutical Factory, Inc. Tokyo, Japan). All efforts were made to minimize suffering.

Rats were fixed on their back on an experimental stand. After the experiment, all the rats were sacrificed using CO_2_ gas.

Each solution (physiological saline [PS], *E*. *coli*-LPS [5 mg/mL], and *E*. *coli*-LPS [5 mg/mL]/EA [15 *μ*g/mL]) were topically applied into the right or left gingival sulcus of the maxillary molars every 10 minutes for 1 hour (six times, 2 *μ*L each time). Periodontal tissue samples were subsequently obtained on day 3, following topical application, for immunohistochemistry. In this model, an increase in the osteoclasts on the surface of the alveolar bone toward the periodontal ligament was confirmed at 3 days following LPS-application [36]. Tissue preparation was performed as previously described by Yamano et al. [37]. To count the number of osteoclasts formed along the alveolar bone surface, 4.5 *μ*m sections, including the root from the alveolar crestal area to the root apex area, were prepared from the periodontal tissue around the first and second molars and stained with hematoxylin and eosin (H&E) for routine histological evaluation.

### 2.7. Immunohistochemistry

Immunohistochemical staining was performed as previously described by Furusho et al. [38]. After dewaxing and rehydration, the 4.5 *μ*m sections were incubated in 0.3% hydrogen peroxide in methanol for 30 minutes at room temperature (range: 22 ± 5°C) to quench the endogenous peroxidase activity. After incubation with a protein block (DAKO Japan, Tokyo, Japan) for 10 minutes at room temperature, immunolocalization of RANKL, OPG, and cathepsin K was detected using the anti-rat sRANKL rabbit antibody (#ab62516; 1 : 2000 dilution, Abcam, CB, UK) or anti-rat OPG rabbit antibody (#ab73400; 1 : 400 dilution, Abcam, CB, UK) or anti-rat cathepsin K rabbit antibody (#ab19027; 1 : 500 dilution, Abcam, CB, UK). Anti-rabbit IgG antibody (EnVision^+^ System- HRP Labelled Polymer Anti-Rabbit, Dako Japan) was used as a secondary antibody. After washing the sections twice in phosphate-buffered saline (PBS) for 5 minutes each, staining was visualized using the DAB peroxidase (HRP) substrate kit (Dako Japan) to produce brown reaction products indicative of antigen localization.

### 2.8. Histomorphometrical Analysis of Number of Osteoclasts

**Figure 1:**
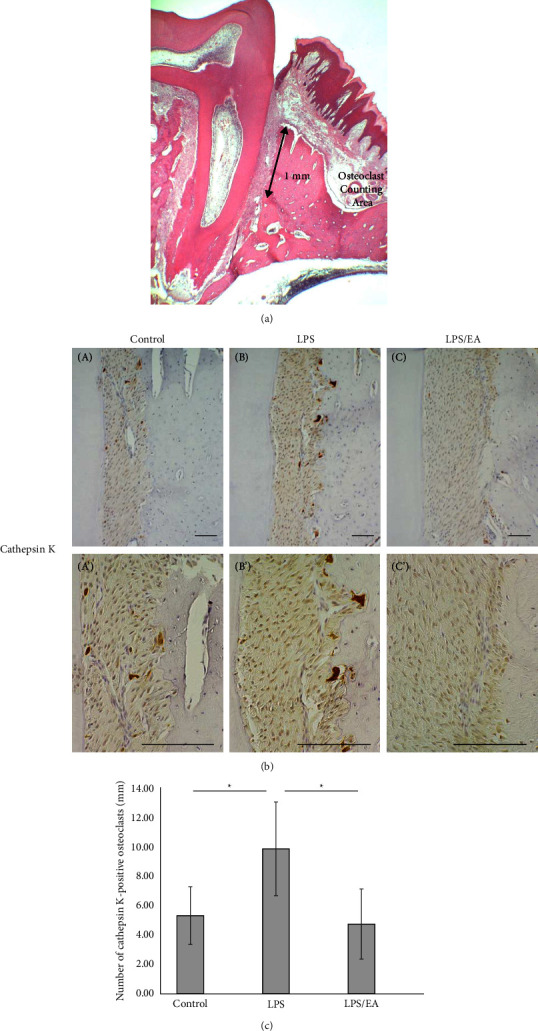
Effect of *Equisetum arvense* extract (EA) on lipopolysaccharide (LPS)-induced osteoclast formation. Effects of EA (15 *μ*g/mL) on osteoclast formation by *Escherichia coli* (*E*. *coli*)-LPS (5 mg/mL). Periodontal tissue was collected 3 days following treatment, and immunohistochemical (IHC) staining was performed. (a) Illustration of the osteoclast counting area. (b) Immunoexpression of cathepsin K, an osteoclast marker after 3 days following (A, A′) physiological saline (control), (B, B′) *E*. *coli*-LPS, and (C, C′) *E*. *coli*-LPS/EA application. Scale bars = 100 *μ*m. (c) The number of cathepsin K-positive osteoclasts formed along the alveolar bone margin within 1 mm of the alveolar crest is counted. Data are presented as means ± standard deviation (*n* = 7 for each group). Tukey–Kramer multiple comparison test, ^*∗*^*p* < 0.05.

For the histomorphometric evaluation of the osteoclasts, more than 12 representative sections containing the root apex of the disto-palatal roots of the right and left upper first and second molars from each experimental group were selected. Tissue sections stained with cathepsin K were photographed at 40× and cathepsin K-positive osteoclasts formed along the alveolar bone margin contained within 1 mm from the alveolar crest were counted manually (Figure 1(a)) to evaluate the LPS-induced osteoclast formation. The mean value of the first and second molars was used as the number of osteoclasts per sample.

### 2.9. Statistical Analysis

Results are reported as the mean ± standard deviation. Intergroup differences were compared using the Tukey–Kramer multiple comparisons test, which was conducted using the multcomp package in *R* [39]. Statistical significance was set at *p* < 0.05 or *p* < 0.01 or *p* < 0.001.

## 3. Results

### 3.1. Topical Administration of EA Decreases Osteoclasts in the Alveolar Bone

The inhibitory effect of EA on osteoclastogenesis was analyzed using the rat model of LPS-induced periodontitis, in which the time changes in tissue destruction, including neutrophil migration, osteoclastogenesis, and cytokine expression, are well established [40].

Osteoclasts are multinucleated cells that are differentiated from monocytic and macrophage progenitors and are the only cells capable of resorbing calcified bone. Cathepsin K is a papain-like cysteine protease member of the cathepsin family and is only expressed at high levels in osteoclasts. Cathepsin K is reportedly present in lysosomes and intracytoplasmic vesicles along the osteoclast-bone resorption interface in osteoclasts [41].  Figure 1(b) shows the immunolocalization of cathepsin K in each experimental group. In the PS-applied control group, a few cathepsin K positive osteoclasts, which were small-sized and located further from the alveolar bone margin, were observed (Figures 1(b) A and A′). LPS application induced numerous cathepsin K positive multinucleated osteoclasts in Howship's lacunae along the alveolar bone margin (Figures 1(b) B and B′). In the *E*. *coli*-LPS/EA groups, the number of osteoclasts was markedly reduced (Figures 1(b) C and C′).

Figure 1(c) shows the number of cathepsin K-positive osteoclasts that appeared along the alveolar bone margin in an area 1 mm from the alveolar bone crest (Figure 1(a)). The number of cathepsin K-positive osteoclasts significantly increased in the LPS group compared to the control group (*p* < 0.05). Conversely, EA application significantly reduced the LPS-induced osteoclasts to the level of the control group (*p* < 0.05).

### 3.2. EA Maintains a Balance in the RANKL/OPG Ratio in the Periodontal Ligament

**Figure 2:**
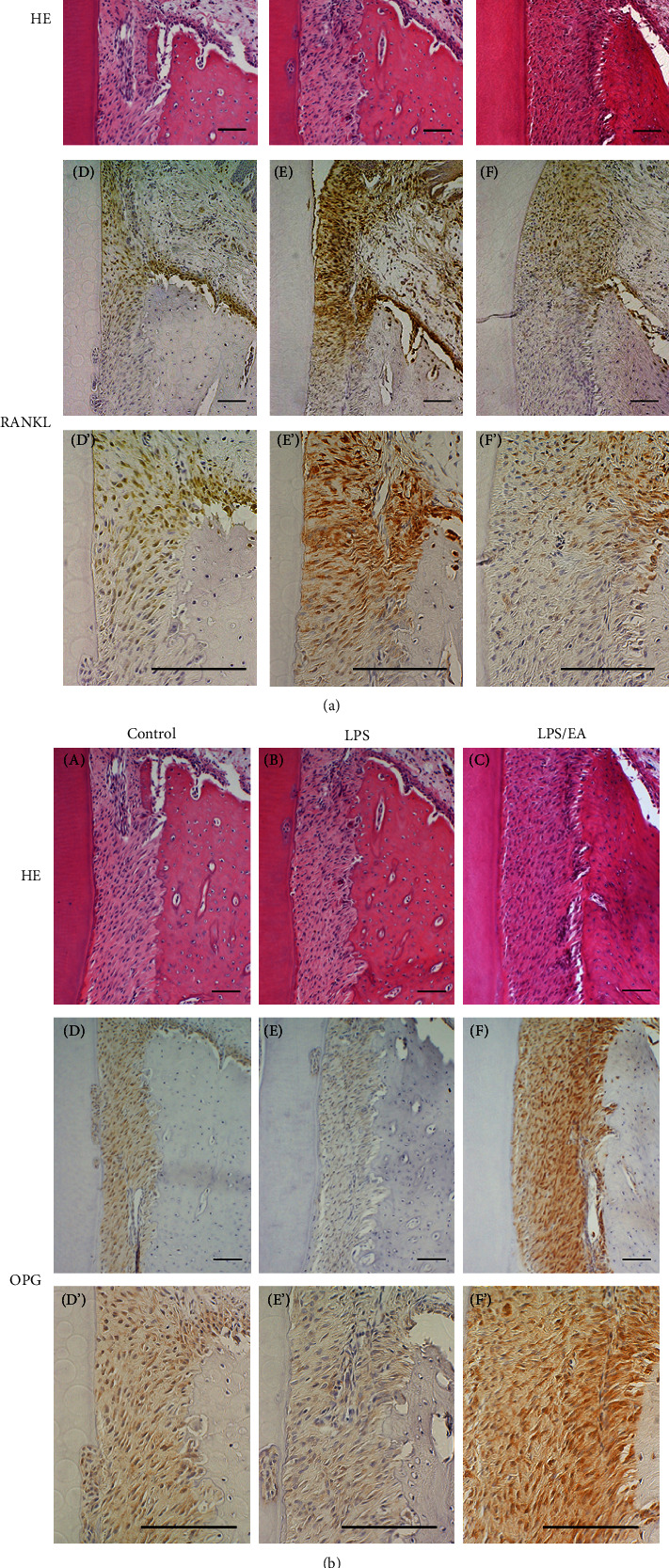
Immunohistochemical examination of periodontal tissue. (a) Hematoxylin and eosin (H&E) staining of tissue obtained from animals 3 days after *Escherichia coli* (*E. coli*)-lipopolysaccharide (LPS) topical administration. (A) Control (PS-applied control), (B) *E*. *coli*-LPS, (C) *E*. *coli*-LPS/*Equisetum arvense* extract (EA) application. Immunoexpression of the receptor activator of NF-*κ*B ligand (RANKL) in the upper part of the alveolar bone area, (D, D′) control, (E, E′) *E*. *coli*-LPS, and (F, F′) *E*. *coli*-LPS/EA application. Scale bars = 100 *μ*m. (b) H&E staining of tissue obtained from animals 3 days after *E*. *coli*-LPS topical administration. (A) Control, (B) *E*. *coli*-LPS, and (C) *E*. *coli*-LPS/EA application. Immunoexpression of osteoprotegerin (OPG) in the upper part of the alveolar bone area, (D, D′) control, (E, E′) *E*. *coli*-LPS, and (F, F′) *E*. *coli*-LPS/EA application. Scale bars = 100 *μ*m.

A balance in RANKL/OPG plays an essential role in determining the level of osteoclastogenesis. In the present study, immunohistochemical staining was performed to verify the expression levels of RANKL and OPG (Figure 2). In the control group, strong RANKL positivity was demonstrated in the osteoblastic cells in the periosteum, and weak RANKL positive cells were scattered in the periodontal ligament of the alveolar crestal area (Figures 2(a) D and D′). In contrast, in the LPS group, an intensely positive reaction was induced in the fibroblasts of the periodontal ligament at the alveolar crestal area (Figures 2(a) E and E′). However, in the *E. coli*-LPS/EA group, the expression of the RANKL protein was markedly reduced (Figures 2(a) F and F′).

Weak OPG-positive reaction was constitutively detected in the fibroblasts of the periodontal ligament and osteoblastic cells in the periosteum in the control group (Figures 2(b) D and D′). The expression of OPG was reduced in the LPS group (Figures 2(b) and E′). Interestingly, in the *E*. *coli*-LPS/EA group, the expression of OPG recovered and tended to be more enhanced than that of the control group (Figures 2(b) F and F′). Moreover, in the control group, the RANKL expression was strong in the upper part of the alveolar bone; however, OPG was also expressed in this area, and osteoclastogenesis was not induced since the balance in RANKL/OPG was maintained (Figures 2(a) D and D′).

In the control group, the expression of RANKL is suppressed to a low level, and OPG is expressed (Figures 2(a) D and 2(b) D). In the LPS-administered group, RANKL levels in the periodontal ligament fibroblasts and osteoblasts have increased, but the expression of OPG has suppressed (Figures 2(a) E and 2(b) E). In contrast, in the *E*. *coli*-LPS/EA-administered group, RANKL is suppressed, while the OPG expression is restored, and the staining intensity is higher than that of the control expression (Figures 2(a) F and 2(b) F).

### 3.3. EA Inhibited LPS-Induced Changes in the Factors Regulating Osteoclast Formation

Osteoclast differentiation and function are closely regulated by the osteoblasts and other cells of the osteoblast lineage. To investigate the effect of EA on the expression of the factors regulating osteoclast formation in the ST2 cells with LPS stimulation, the mRNA expression of IL-6, TNF-*α*, IL-1*β*, RANKL, OPG, and Sema3A was examined by quantitative real-time PCR.

**Figure 3:**
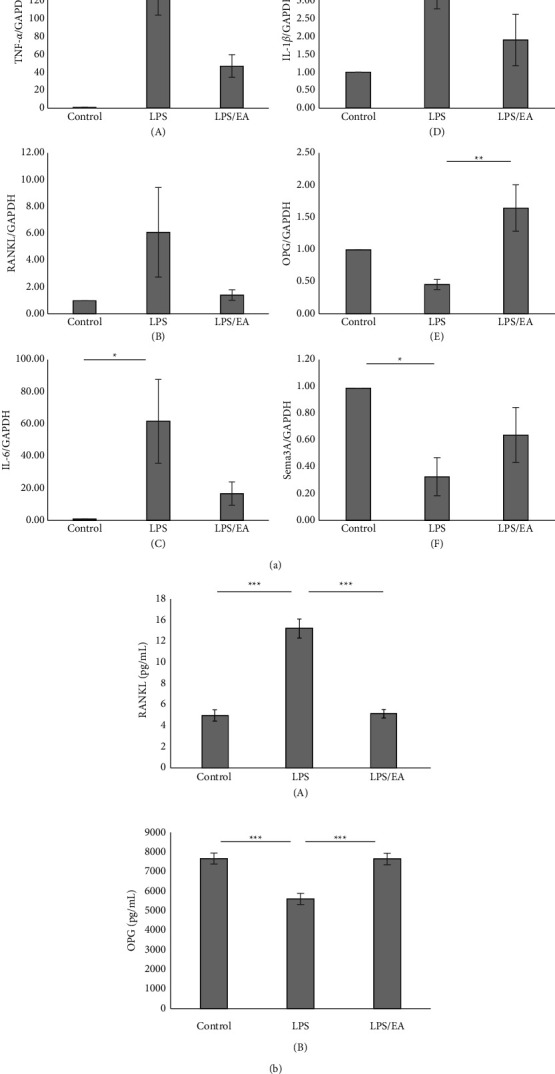
Effect of *Equisetum arvense* extract (EA) on osteoclast formation-related factors. (a) It has been reported that stimulation of osteoblasts with lipopolysaccharide (LPS) alters the expression of osteoclast formation-related factors. Therefore, we examined the effect of EA on osteoclast formation-related factors. Tumor necrosis factor-*α*, interleukin (IL)-6, and the receptor activator of NF-*κ*B ligand (RANKL) mRNA expression of ST2 stimulated with *Escherichia coli* (*E*. *coli*)-LPS (1 *μ*g/mL) with or without EA (3 *μ*g/mL) for 2 hours, and total RNAs are extracted (A, B, C). IL-1*β* and Sema3A mRNA expression of ST2 stimulated with *E*. *coli*-LPS with or without EA for 12 hours, and total RNAs are extracted (D, F). Osteoprotegerin (OPG) mRNA expression of ST2 stimulated with *E*. *coli*-LPS with or without EA for 24 hours, and total RNAs are extracted (E). Glyceraldehyde 3-phosphate dehydrogenase (GAPDH) is used as an internal control. Data are presented as means ± standard error (*n* = 7 for each group). Tukey–Kramer multiple comparison test, ^*∗∗∗*^*p* < 0.001, ^*∗∗*^*p* < 0.01, ^*∗*^*p* < 0.05. (b) RANKL and OPG protein expression of ST2 stimulated with *Escherichia coli* (*E*. *coli*)-lipopolysaccharide (LPS) (1 *μ*g/mL). Cell culture supernatants were collected 3 hours (for RANKL) or 48 hours (for OPG) after treatment and analyzed using an ELISA assay. Data are presented as means ± standard error (*n* = 6 for each group). Tukey–Kramer multiple comparison test, ^*∗∗∗*^*p* < 0.001.

The results demonstrated that EA significantly inhibited TNF-*α* mRNA expression attributed to LPS stimulation (Figure 3(a) A) and revealed the inhibitory tendency of RANKL, IL-6, and IL-1*β* mRNA expression (Figures 3(a) B–D).

On the other hand, EA significantly upregulated the OPG mRNA (Figure 3(a) E) expression suppressed by LPS stimulation. Moreover, LPS-induced inhibition of Sema3A mRNA (Figure 3(a) F) showed a recovery tendency.

Subsequently, we examined the effects of EA on *E*. *coli*-LPS induced RANKL protein secretion in ST2 cells by the enzyme-linked immunoassay (ELISA) system. The results showed that EA significantly suppressed the RANKL protein secretion (Figure 3(b) A). Moreover, EA significantly increased the OPG secretion reduced by LPS stimulation (Figure 3(b) B).

### 3.4. EA Inhibited LPS-Induced Phosphorylation of IKK, NF-*κ*B in ST2

**Figure 4:**
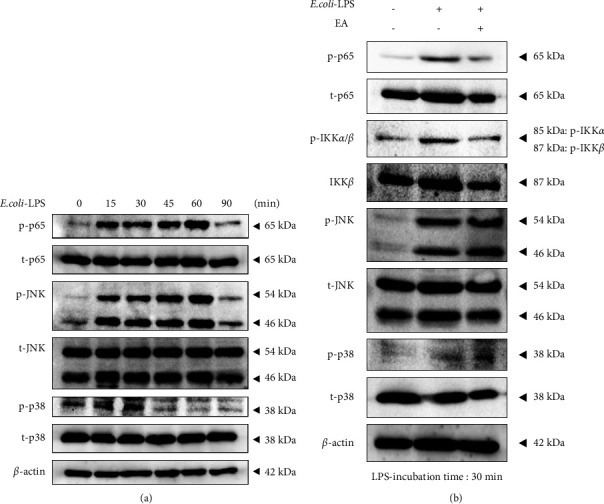
Effects of EA on the LPS induced NF-*κ*B and MAPKs' signaling pathway in ST2. (a) *E. coli*-LPS (1 *μ*g/mL) is applied to ST2 cells and collected at several time points. NF-*κ*B p65 and MAPKs (JNK and p38) in ST2 cells are analyzed by Western blotting. *β*-actin is used as a loading control. (b) EA: *Equisetum arvense* extract (3 *μ*g/mL) is pretreated in ST2 cells for 30 minutes, and then *E*. *coli*-LPS (1 *μ*g/mL) is additionally applied to ST2 cells. IKK*α*/*β*, NF-*κ*B p65, and MAPKs (JNK and p38) in ST2 cells are assessed at 30 minutes by Western blotting. *β*-actin is used as the loading control.

Nuclear factor-*κ*B (NF-*κ*B) and MAPKs play important roles in inflammatory cytokine production caused by LPS-TLR4 signaling [42, 43]. Since our results showed that EA reduced LPS-stimulated expression of inflammatory cytokines, we suggest that EA may also inhibit phosphorylation of NF-*κ*B and MAPKs; c-Jun*N*-terminal kinase (JNK) and p38, which are upstream of inflammatory cytokines. We investigated the effects of EA on these signaling pathways caused by *E*. *coli*-LPS (1 *μ*g/mL) by Western blotting. The time course analysis showed that *E*. *coli*-LPS induced activation of NF-*κ*B p65, JNK, and p38 during 15–60 minutes, decreasing after 90 minutes (Figure 4(a)). Subsequently, the effects of EA on the phosphorylation of I*k*B kinase alpha, I*k*B kinase beta (IKK*α*/*β*), NF-*κ*B p65, JNK, and p38 are examined at 30 minutes after LPS stimulation. EA strongly downregulated p-IKK*α*/*β* and p-NF-*κ*B p65 activated by *E*. *coli*-LPS but did not downregulate pJNK or p-p38 (Figure 4(b)).

### 3.5. EA Inhibited LPS-Induced Degradation of *β*-Catenin in ST2

**Figure 5:**
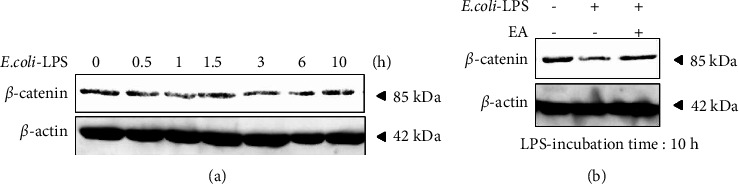
Effects of EA on the LPS induced downregulation of Wnt/*β*-catenin signaling in ST2. (a) *E. coli*-LPS (1 *μ*g/mL) is applied to ST2 cells and collected at several time points. *β*-Catenin in ST2 cells is analyzed by Western blotting. *β*-actin is used as a loading control. (b) *E*. *coli*-LPS (1 *μ*g/mL) and/or EA; *Equisetum arvense* extract (3 *μ*g/mL) are treated in ST2 cells for 10 hours on ST2 cells. *β*-Catenin in ST2 cells is analyzed by Western blotting. *β*-actin is used as the loading control.

Since OPG expression is regulated by Wnt/*β*-catenin signaling pathways in osteoblasts [44], it was suggested that EA increased the OPG reduced by LPS stimulation via the Wnt/*β*-catenin signaling pathway. Therefore, we investigated the effects of EA on the Wnt/*β*-catenin signaling pathways caused by *E*. *coli*-LPS (1 *μ*g/mL) by Western blotting. The time course analysis shows that *E*. *coli*-LPS induced degradation of *β*-catenin after 30 min upon stimulation with *E*. *coli*-LPS (Figure 5(a)). Subsequently, we examined the effect of EA on the degradation of *β*-catenin by *E*. *coli*-LPS. The results demonstrates that EA inhibited *β*-catenin degradation in ST2 stimulated cells with *E*. *coli*-LPS (Figure 5(b)).

## 4. Discussion

In this study, we confirmed that EA inhibited alveolar bone destruction through the control of osteoblast-mediated osteoclastogenesis by reducing phosphorylated NF-*κ*B, TNF-*α,* and RANKL and upregulating Sema3A, *β*-catenin, and OPG with LPS stimulation. Notably, EA only suppressed the increase in osteoclasts induced by LPS application to the control level, suggesting that EA may not inhibit physiological bone remodeling. This is the first report demonstrating the inhibitory effect of EA on osteoclastogenesis *in vivo*.

Periodontitis is caused by the repeated challenge of harmful irritants, such as LPS, on the periodontal tissues owing to persistent infection by periodontal bacteria resulting in chronic host immune and inflammatory responses and tissue destruction, including alveolar bone resorption. Bone homeostasis is maintained via concerted communication between bone-building osteoblasts, bone-degrading osteoclasts, and osteocytes as the mechanosensory cells of the bone.

EA has osteogenic properties and has been reported to induce human osteoblasts *in vitro* [45]. EA can reportedly promote bone healing following bone surgery and fractures. However, considering the inhibitory effect of EA on osteoclastogenesis, it is reported that EA suppressed osteoclast progenitor cell differentiation caused by the macrophage colony-stimulating factor and RANKL stimulation *in vitro* [34]. Besides, there is no report verifying the effect of EA on bone resorption associated with inflammation *in vivo* We are the first to demonstrate the inhibitory effect of EA on LPS-induced osteoclastogenesis using an animal model.

Osteoclast differentiation and activity are regulated by RANKL and OPG, mainly produced by osteoblast lineage cells. In this study, we found that EA suppressed the production of RANKL, which was enhanced by LPS, and restored the production of OPG downregulated by LPS, using the rat model of periodontitis. Particularly, OPG expression in the periodontal ligament tissue of the *E*. *coli*-LPS/EA group was markedly upregulated in comparison with that of the control group. OPG is a soluble molecule that regulates bone destruction by potently inhibiting osteoclasts.

Since OPG expression is regulated by Wnt/*β*-catenin signaling pathways in osteoblasts [44], we performed Western blotting using osteoblast lineage cells (ST2) to investigate the effect of EA on the upstream signaling of RANKL and OPG induced by LPS.

**Figure 6:**
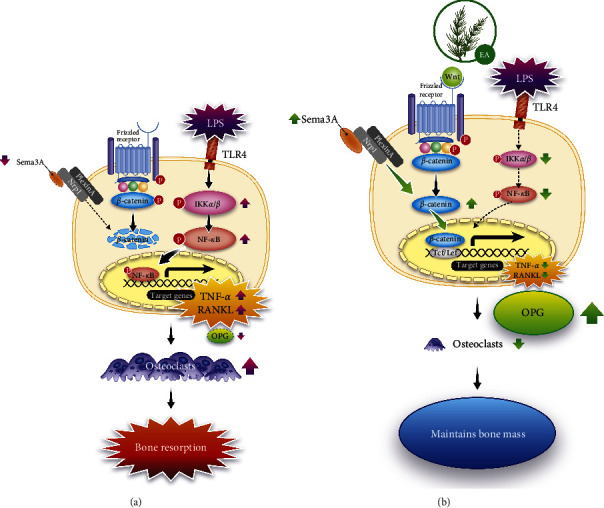
Proposed mechanism of EA inhibition of osteoclast formation caused by LPS. (a) In LPS-induced periodontitis, TLR4-mediated phosphorylation of I*κ*B kinase *α* and *β* (IKK*α*/*β*) and NF-*κ*B induces the expression of inflammatory cytokines such as tumor necrosis factor (TNF)-*α* in periodontal tissue cells and the generation of the osteoclastogenic receptor activator of NF-*κ*B ligand (RANKL), an osteoclastogenic factor. However, it inactivates the Wnt/*β*-catenin signaling pathway and promotes *β*-catenin degradation. Furthermore, production of the secreted protein semaphorin 3A (Sema3A) is decreased; Sema3A/neuropilin-1 (Nrp1)-mediated *β*-catenin degradation and nuclear translocation are suppressed, as is transcription and expression of osteoprotegerin (OPG), the decoy receptor for RANKL. Finally, the RANKL/OPG ratio is increased, leading to osteoclast formation and maturation and subsequent bone resorption. (b) Proposed mechanism of EA-induced suppression of osteoclastogenesis by LPS: EA strongly suppresses the activation of IKK*α*/*β* and NF-*κ*B, indicating that the Wnt/*β*-catenin signaling pathway is activated and *β*-catenin degradation is suppressed. Furthermore, Sema3A production is elevated, Sema3A/Nrp1-mediated *β*-catenin degradation is suppressed, and nuclear migration is promoted. This suppresses osteoclast formation and excessive bone resorption.

Figure 6 shows a schematic representation of the proposed mechanism by which EA suppresses osteoclastogenesis by LPS: LPS induces activation of IKK*α*/*β* and its downstream NF-*κ*B via TLR4, upregulates TNF-*α* and RANKL expression, and inactivates the Wnt/*β*-catenin signaling pathway, leading to downregulation of OPG expression. Furthermore, the production of the secreted protein Sema3A is reduced, inhibiting neuropilin-1-mediated *β*-catenin degradation and nuclear translocation and suppressing OPG expression as well (Figure 6(a)). In contrast, EA strongly suppresses IKK*α*/*β* and NF-*κ*B activation caused by LPS stimulation, resulting in reduction of RANKL.

While OPG is upregulated through activating the Wnt/*β*-catenin signaling pathway and promoting Sema3A/neuropilin-1-mediated nuclear migration of *β*-catenin. Thus, EA suppresses osteoclast formation and excessive bone resorption caused by LPS (Figure 6(b)).

Several proinflammatory cytokines enhance osteoclast differentiation and function. For example, TNF-*α* promotes the increase of osteoclast progenitor cells *in vivo* [30–33]. Furthermore, IL-1*α* and *β* directly act on the osteoclasts without RANKL to prolong the osteoclast life and activate bone resorption [46]. Inflammatory cytokines can also affect bone formation. For instance, TNF-*α* acts in an inhibitory manner on osteoblast differentiation and survival; TNF-*α* reportedly suppresses the production of insulin-like growth factor 1, which acts to increase the number of osteoblast progenitor cells [47], and suppresses the transcription of runt-related transcription factor 2, a master transcription factor for osteoblast differentiation [48]. Furthermore, TNF-*α* and IL-1*β* have been reported to act on osteoblasts to increase Fas expression and induce apoptosis [49]. Previously, by using the rat model of LPS-induced periodontitis, we confirmed that EA treatment markedly reduced immunoexpression of TNF-*α* at the junctional epithelium [29]. In the present study, evaluation using osteoblast lineage cells demonstrated the strong inhibitory effect of EA on *E*. *coli*-LPS-induced production of inflammatory cytokines (TNF-*α*, IL-1*β*, and IL-6). These findings suggest that EA may strongly inhibit osteoclastogenesis by restoring OPG production and suppressing the production of inflammatory cytokines and RANKL induced by LPS.

These effects of EA could be attributed to the compounds contained in *E. arvense*. For instance, quercetin in *E. arvense* has been shown to inhibit the formation, proliferation, and maturation of osteoclasts [50, 51]. Osteoblast-like cells with quercetin treatment reportedly show an increased tendency in OPG production and a significant decrease in RANKL [52, 53]. Moreover, quercetin has an effect of promoting mature osteoclast apoptosis [50, 54].

In the present study, we focused on the inhibitory effect of EA on bone destruction; however, positive effects are also expected in osteogenesis. The current *in vitro* study demonstrated that EA restored the LPS-induced downregulation of Sema3A and *β*-catenin, which is homeostatically produced by osteoblast lineage cells. Sema3A inhibits osteoclast differentiation via neuropilin-1 on osteoclast progenitor cells, which also acts on osteoblasts themselves, promoting osteoblast differentiation through the Wnt/*β*-catenin signaling pathway [55]. *E. arvense* contains several compounds that reportedly promote osteoblast formation. Ursolic acid contained in *E. arvense* has been shown to promote osteoblast differentiation and mineralization *in vitro* [26]. Furthermore, silica, rich in *E. arvense*, promotes the deposition of calcium and other minerals, decreases the number of osteoclasts, stimulates the activity of osteoblasts, promotes the synthesis of collagen, and facilitates the synthesis of glycosaminoglycans and collagen, resulting in the formation of bone and connective tissue [28, 56]. This evidence indicates that the compounds in EA promote osteoblast formation; therefore, EA is expected to have the effect of not only inhibiting osteoclast formation but also promoting bone formation by osteoblasts *in vivo*. The osteogenic activity of EA should be verified in future studies.

Antiresorptive and anabolic agents are effective treatments for osteoclastic lesions; however, some side effects have been reported. For instance, antiresorptive osteonecrosis of the jaw caused by anti-RANKL antibodies and bisphosphonates has become a major problem [57]. Hormone-replacement therapy can attribute to an increased risk of venous thromboembolism and cardiovascular events [58, 59]. A prolonged treatment period and high administration dose of recombinant human parathyroid hormone (teriparatide) increases the incidence of bone neoplasms [60]. Therefore, natural compounds such as EA with potent osteoprotective properties and few side effects could be an alternative strategy to overcome the shortcomings of existing treatments for osteoclastic lesions as well as to develop oral care methods to prevent the destruction of alveolar bone associated with periodontitis.

## 5. Conclusion

The main limitation of this study was the use of *E*. *coli*-LPS for the animal experiment. Although we should have used LPS from periodontal pathogen, we chose this model because of the established time course of histological changes in periodontitis caused by *E*. *coli*-LPS administration. *E*. *coli*-LPS is known to be a type of *Aggregatibacter actinomycetemcomitans*-LPS, which is one of the main periodontal pathogens; however, the effects of EA on LPS from periodontal pathogen-induced periodontal changes *in vivo* should be clarified in future studies.

This study suggests that EA could be useful in preventing or treating periodontal disease and other bone-destroying lesions. Considering the clinical application, the daily use of toothpaste and mouthwash containing EA could inhibit alveolar bone resorption associated with plaque accumulation in periodontal pockets and suppress tooth mobility and tooth loss attributed to periodontitis, eventually resulting in the maintenance of healthy teeth for a lifetime.

## Data Availability

All data are available from the corresponding author upon reasonable request.

## References

[1] Tanabe N., Maeno M., Suzuki N. (2005). IL-1*α* stimulates the formation of osteoclast-like cells by increasing M-CSF and PGE2 production and decreasing OPG production by osteoblasts. *Life Sciences*.

[2] Wang H. y, Qin H. x, Deng H., Sun C. f, Hu R. d (2015). Effects of LPS-stimulated monocyte culture supernatant on the OPG/RANKL of osteoblastic cells. *Shanghai Journal of Stomatology*.

[3] Chuang F. H., Tsai C. C., Chen J. H., Chen K. K., Chen Y. K., Lin Y. C. (2012). Long-term sequential receptor activator of NF-*κ*B ligand (RANKL) and osteoprotegrin (OPG) expression in lipopolysaccharide-induced rat periapical lesions. *Journal of Oral Pathology & Medicine*.

[4] Yu X. J., Xiao C. J., Du Y. M., Liu S., Du Y., Li S. (2015). Effect of hypoxia on the expression of RANKL/OPG in human periodontal ligament cells in vitro. *International Journal of Clinical and Experimental Pathology*.

[5] Yu X., Lv L., Zhang J., Zhang T., Xiao C., Li S. (2015). Expression of neuropeptides and bone remodeling-related factors during periodontal tissue regeneration in denervated rats. *Journal of Molecular Histology*.

[6] Heitz-Mayfield L. J. A., Lang N. P. (2013). Surgical and nonsurgical periodontal therapy. Learned and unlearned concepts. *Periodontology 2000*.

[7] Kressin N. R., Boehmer U., Nunn M. E., Spiro A. (2003). Increased preventive practices lead to greater tooth retention. *Journal of Dental Research*.

[8] Do Monte F. H. M., dos Santos J. G., Russi M., Bispo Lanziotti V. M. N., Leal L. K. A. M., de Andrade Cunha G. M. (2004). Antinociceptive and anti-inflammatory properties of the hydroalcoholic extract of stems from Equisetum arvense L. in mice. *Pharmacological Research*.

[9] Asgarpanah J., Roohi E. (2012). Phytochemistry and pharmacological properties of Equisetum arvense L. *Journal of Medicinal Plants Research*.

[10] Stajner D., Popović B. M., Canadanović-Brunet J., Anackov G. (2009). Exploring Equisetum arvense L., Equisetum ramosissimum L. and Equisetum telmateia L. as sources of natural antioxidants. *Phytotherapy Research*.

[11] Milovanović V., Radulović N., Todorović Z., Stanković M., Stojanović G. (2007). Antioxidant, antimicrobial and genotoxicity screening of hydro-alcoholic extracts of five Serbian Equisetum species. *Plant Foods for Human Nutrition*.

[12] Dos Santos J. G., Blanco M. M., Do Monte F. H. (2005). Sedative and anticonvulsant effects of hydroalcoholic extract of Equisetum arvense. *Fitoterapia*.

[13] Oh H., Kim D. H., Cho J. H., Kim Y. C. (2004). Hepatoprotective and free radical scavenging activities of phenolic petrosins and flavonoids isolated from Equisetum arvense. *Journal of Ethnopharmacology*.

[14] Radulović N., Stojanović G., Palić R. (2006). Composition and antimicrobial activity of Equisetum arvense L. essential oil. *Phytotherapy Research*.

[15] Ganeva Y., Chanev C., Dentchev T. (2001). Triterpenoids and sterols from Equisetum arvense. *Dokladi Na Bulgarskata Akademiya Na Naukite*.

[16] Gründemann C., Lengen K., Sauer B., Garcia-Käufer M., Zehl M., Huber R. (2014). Equisetum arvense (common horsetail) modulates the function of inflammatory immunocompetent cells. *BMC Complementary and Alternative Medicine*.

[17] Prouillet C., Mazière J. C., Mazière C., Wattel A., Brazier M., Kamel S. (2004). Stimulatory effect of naturally occurring flavonols quercetin and kaempferol on alkaline phosphatase activity in MG-63 human osteoblasts through ERK and estrogen receptor pathway. *Biochemical Pharmacology*.

[18] Guo A. J., Choi R. C., Zheng K. Y. (2012). Kaempferol as a flavonoid induces osteoblastic differentiation via estrogen receptor signaling. *Chinese Medicine*.

[19] Pang J. L., Ricupero D. A., Huang S. (2006). Differential activity of kaempferol and quercetin in attenuating tumor necrosis factor receptor family signaling in bone cells. *Biochemical Pharmacology*.

[20] Wattel A., Kamel S., Prouillet C. (2004). Flavonoid quercetin decreases osteoclastic differentiation induced by RANKL via a mechanism involving NF kappa B and AP-1. *Journal of Cellular Biochemistry*.

[21] Choi E. M. (2007). Apigenin increases osteoblastic differentiation and inhibits tumor necrosis factor-alpha-induced production of interleukin-6 and nitric oxide in osteoblastic MC3T3-E1 cells. *Pharmazie*.

[22] Bandyopadhyay S., Lion J. M., Mentaverri R. (2006). Attenuation of osteoclastogenesis and osteoclast function by apigenin. *Biochemical Pharmacology*.

[23] Kim T. H., Jung J. W., Ha B. G. (2011). The effects of luteolin on osteoclast differentiation, function in vitro and ovariectomy-induced bone loss. *The Journal of Nutritional Biochemistry*.

[24] Bian Q., Liu S. F., Huang J. H. (2012). Oleanolic acid exerts an osteoprotective effect in ovariectomy-induced osteoporotic rats and stimulates the osteoblastic differentiation of bone mesenchymal stem cells in vitro. *Menopause*.

[25] Lo Y. C., Chang Y. H., Wei B. L., Huang Y. L., Chiou W. F. (2010). Betulinic acid stimulates the differentiation and mineralization of osteoblastic MC3T3-E1 cells: involvement of BMP/Runx2 and *β*-Catenin signals. *Journal of Agricultural and Food Chemistry*.

[26] Lee S. U., Park S. J., Kwak H. B., Oh J., Min Y. K., Kim S. H. (2008). Anabolic activity of ursolic acid in bone: stimulating osteoblast differentiation in vitro and inducing new bone formation in vivo. *Pharmacological Research*.

[27] Currie H. A., Perry C. C. (2009). Chemical evidence for intrinsic ‘Si’ within Equisetum cell walls. *Phytochemistry*.

[28] Holzhüter G., Narayanan K., Gerber T. (2003). Structure of silica in Equisetum arvense. *Analytical and Bioanalytical Chemistry*.

[29] Shiba F., Miyauchi M., Chea C. (2021). Anti-inflammatory effect of glycyrrhizin with Equisetum arvense extract. *Odontology*.

[30] Anandarajah A. P., Schwarz E. M., Totterman S. (2007). The effect of etanercept on osteoclast precursor frequency and enhancing bone marrow oedema in patients with psoriatic arthritis. *Annals of the Rheumatic Diseases*.

[31] Yao Z., Li P., Zhang Q. (2006). Tumor necrosis factor-alpha increases circulating osteoclast precursor numbers by promoting their proliferation and differentiation in the bone marrow through up-regulation of c-Fms expression. *Journal of Biological Chemistry*.

[32] Li P., Schwarz E. M., O’Keefe R. J. (2004). Systemic tumor necrosis factor *α* mediates an increase in peripheral CD11b^high^ osteoclast precursors in tumor necrosis factor *α*-transgenic mice. *Arthritis & Rheumatism*.

[33] Zhang Q., Guo R., Schwarz E. M., Boyce B. F., Xing L. (2008). TNF inhibits production of stromal cell-derived factor 1 by bone stromal cells and increases osteoclast precursor mobilization from bone marrow to peripheral blood. *Arthritis Research and Therapy*.

[34] Costa-Rodrigues J., Carmo S. C., Silva J. C., Fernandes M. H. R. (2012). Inhibition of human in vitro osteoclastogenesis by Equisetum arvense. *Cell Proliferation*.

[35] Kudo Y., Takata T., Ogawa I. (2000). p27Kip1 accumulation by inhibition of proteasome function induces apoptosis in oral squamous cell carcinoma cells. *Clinical Cancer Research*.

[36] Oka H., Miyauchi M., Furusho H., Nishihara T., Takata T. (2012). Oral administration of prostaglandin E(2)-specific receptor 4 antagonist inhibits lipopolysaccharide-induced osteoclastogenesis in rat periodontal tissue. *Journal of Periodontology*.

[37] Yamano E., Miyauchi M., Furusyo H. (2010). Inhibitory effects of orally administrated liposomal bovine lactoferrin on the LPS-induced osteoclastogenesis. *Laboratory Investigation*.

[38] Furusho H., Miyauchi M., Hyogo H. (2013). Dental infection of Porphyromonas gingivalis exacerbates high fat diet-induced steatohepatitis in mice. *Journal of Gastroenterology*.

[39] Hothorn T., Bretz F., Westfall P. (2008). Simultaneous inference in general parametric models. *Biometrical Journal*.

[40] Miyauchi M., Sato S., Kitagawa S. (2001). Cytokine expression in rat molar gingival periodontal tissues after topical application of lipopolysaccharide. *Histochemistry and Cell Biology*.

[41] Littlewood-Evans A., Kokubo T., Ishibashi O. (1997). Localization of cathepsin K in human osteoclasts by in situ hybridization and immunohistochemistry. *Bone*.

[42] Rebaï O., Le Petit-Thevenin J., Bruneau N., Lombardo D., Vérine A. (2005). In vitro angiogenic effects of pancreatic bile salt-dependent lipase. *Arteriosclerosis, Thrombosis, and Vascular Biology*.

[43] Dai J., Peng L., Fan K. (2009). Osteopontin induces angiogenesis through activation of PI3K/AKT and ERK1/2 in endothelial cells. *Oncogene*.

[44] Glass D. A., Bialek P., Ahn J. D. (2005). Canonical Wnt signaling in differentiated osteoblasts controls osteoclast differentiation. *Developmental Cell*.

[45] Bessa Pereira C., Gomes P. S., Costa-Rodrigues J. (2012). Equisetum arvense hydromethanolic extracts in bone tissue regeneration: in vitro osteoblastic modulation and antibacterial activity. *Cell Proliferation*.

[46] Jimi E., Nakamura I., Duong L. T. (1999). Interleukin 1 induces multinucleation and bone-resorbing activity of osteoclasts in the absence of osteoblasts/stromal cells. *Experimental Cell Research*.

[47] Gilbert L., He X., Farmer P. (2000). Inhibition of osteoblast differentiation by tumor necrosis factor-*α*. *Endocrinology*.

[48] Gilbert L., He X., Farmer P. (2002). Expression of the osteoblast differentiation factor RUNX2 (Cbfa1/AML3/Pebp2*α*A) is inhibited by tumor necrosis factor-*α*. *Journal of Biological Chemistry*.

[49] Tsuboi M., Kawakami A., Nakashima T. (1999). Tumor necrosis factor-*α* and interleukin-1*β* increase the Fas-mediated apoptosis of human osteoblasts. *The Journal of Laboratory and Clinical Medicine*.

[50] Guo C., Hou G. Q., Li X. D. (2012). Quercetin triggers apoptosis of lipopolysaccharide (LPS)-induced osteoclasts and inhibits bone resorption in RAW264.7 cells. *Cellular Physiology and Biochemistry*.

[51] Forte L., Torricelli P., Boanini E. (2016). Antioxidant and bone repair properties of quercetin-functionalized hydroxyapatite: an in vitro osteoblast-osteoclast-endothelial cell co-culture study. *Acta Biomaterialia*.

[52] Zhou Y., Wu Y., Ma W. (2017). The effect of quercetin delivery system on osteogenesis and angiogenesis under osteoporotic conditions. *Journal of Materials Chemistry B*.

[53] Srivastava S., Bankar R., Roy P. (2013). Assessment of the role of flavonoids for inducing osteoblast differentiation in isolated mouse bone marrow derived mesenchymal stem cellsin isolated mouse bone marrow derived mesenchymal stem cells. *Phytomedicine*.

[54] Wattel A., Kamel S., Mentaverri R. (2003). Potent inhibitory effect of naturally occurring flavonoids quercetin and kaempferol on in vitro osteoclastic bone resorption. *Biochemical Pharmacology*.

[55] Hayashi M., Nakashima T., Taniguchi M., Kodama T., Kumanogoh A., Takayanagi H. (2012). Osteoprotection by semaphorin 3Asemaphorin 3A. *Nature*.

[56] Badole S., Kotwal S. (2014). Equisetum arvense: ethanopharmacological and phytochemical review with reference to osteoporosis. *International Journal of Pharmaceutical Science and Health Care*.

[57] Khan A. A., Morrison A., Hanley D. A. (2015). Diagnosis and management of osteonecrosis of the jaw: a systematic review and international consensus. *Journal of Bone and Mineral Research*.

[58] Kling J. M., Clarke B. L., Sandhu N. P. (2014). Osteoporosis prevention, screening, and treatment: a review. *Journal of Women’s Health Health (Larchmt)*.

[59] Mosca L., Grady D., Barrett-Connor E. (2009). Effect of raloxifene on stroke and venous thromboembolism according to subgroups in postmenopausal women at increased risk of coronary heart disease. *Stroke*.

[60] Vahle J. L., Long G. G., Sandusky G., Westmore M., Ma Y. L., Sato M. (2004). Bone neoplasms in F344 rats given teriparatide [rhPTH(1-34)] are dependent on duration of treatment and dose. *Toxicologic Pathology*.

